# White noise and neuronal porosome complex: transmission electron microscopic study

**DOI:** 10.15190/d.2014.17

**Published:** 2014-08-19

**Authors:** Mzia G. Zhvania, Tamar Z. Bikashvili, Nadezhda J. Japaridze, Ilia I. Lazrishvili, Mariam Ksovreli

**Affiliations:** Institute of Chemical Biology, Ilia State University, 3/5 K. Cholokashvili Avenue, 0162 Tbilisi, Georgia; I. Beritashvili Center of Experimental Biomedicine, 14 Gotua Street, 0160 Tbilisi, Georgia; New Vision University, 1a, Mikeladze Street, 0159 Tbilisi, Georgia

**Keywords:** white noise, medial geniculate body, colliculus inferior, ultrastructure of synapses, ultrastructure of porosome

## Abstract

In the present electron microscopic study the effect of continuous white noise on the morphology of synapses and neuronal porosome complex (the neurotransmitter-release or secretory machinery) in two subcortical auditory brain regions - colliculus inferior and medial geniculate body in cat, were investigated. Several morphological alterations in some synapses were detected in both subcortical areas. These alterations mainly indicate to the decrease of functional activity of synapses. Rarely important pathological modifications in pre- and post-synaptic regions were detected. In addition to descriptive studies, the morphometric analysis of porosome diameter and depth was performed in colliculus inferior and medial geniculate body. The results revealed that while white noise has no effect on the porosome diameter and depth in colliculus inferior, it provokes significant alterations in the morphology of porosome complex in medial geniculate body. In particular, the significant increase of porosome depth in this nucleus may reflect the alteration in neurotransmission.

## INTRODUCTION

In all secretory cells, cellular cargo, destined for secretion, is packaged and stored within membranous vesicles that transiently dock and establish continuity at the base of cup- or flask-shaped plasma membrane structures, called “porosomes” and neurons are no exception^1-5^. Porosomes are the universal secretory machinery in cells, where vesicles transiently fuse to release intravesicular content to the outside during secretion. It is suggested that in each type of secretory cell, unique content, different speed of release and different volume of content release dictate specific shape and size of porosomes. Electron microscopy (EM) and atomic force microscopic (AFM) studies in neurons, demonstrate

porosomes to range in size from 10 to 20 nm, where 35-50 nm synaptic vesicles are found to dock (In contrast, in a slow secretory cell like acinar cells of the exocrine pancreas, secretory granules measuring approximately 1000 nm in diameter, expel their content following transient fusion at porosomes measuring 100-180 nm)^1-3,6-8^. Furthermore, recent AFM, solution X-ray, EM, electron density and 3D contour mapping studies provided a large volume of additional information on the nanoscale structure and assembly of the neuronal porosome complex. Thus, it becomes clear that neuronal porosome possessesa central plug, unique structure, that is absent in porosomes in other kind of secretory cells and which works as a peculiar gatekeeper during neurotranmisssion. Specifically, the central plug in neuronal porosome interacts with proteins at the periphery of the structure conforming to the eightfold symmetry; each of them is connected with spoke-like elements to the central plug that is involved in the rapid opening and closing of the neuronal porosome to the outside. Thus, the central plug at various conformations – fully pushed outward (close conformation), halfway retracted and completely retracted (open conformation) into porosome cup has been described^9,10^. Although it is easy to observe porosome in intact synaptosome and in inside-out synaptosome using AFM, using EM it becomes difficult, owing to artifacts producing by fixation, dehydration and other tissue processing for EM. Additional difficulty is encountered due to the content of high density of plasma membrane proteins at the presynaptic membrane resulting in heavy metal staining, rendering difficult to distinguish the porosome from other structures. Nonetheless, some peculiarities of fine morphology of porosome complex are clearly identifiable on EM micrographs in several studies^3-7,11-14^.

In our EM studies we elucidate the neuronal porosome complex in healthy and disease conditions in a number of mammalian species. Thus, the same morphology of porosome, including its similar size, in the “normal” brain of different mammals – rat, cat, and dog – was described earlier^5,12-14^. In all three mammals different conformations of the central plug (open, partially open, closed) were shown, confirming recent view, that despite the fact that porosome is a permanent structure at the presynaptic plasma membrane, it is a highly dynamic^9,10^. The wide range of porosome depth also suggests its dynamic nature^12-14^. Similar heterogeneity of porosome depth is also observed in rats subjected to hypokinetic stress^5,14^. In these animals numerous pathological alterations in the presynaptic terminals and sometimes in postsynaptic regions of the central nucleus of amygdala (brain region involved in the organization of stress-response) have been detected^15^; however, quantitative morphological analysis reveals, that despite such pathologies, there are no significant changes to the porosome structure (diameter and depth). These results however do not exclude the possibility that changes may have occurred in the biochemical profile of the neuronal porosome complex, which remains to be examined. Therefore, we carried out stress studies for longer duration and determine both, morphological and biochemical changes that may occur in neuronal porosome following stress. But it is important to continue the EM investigation of porosome morphology in other pathological and special environmental conditions – conditions which are associated with alterations in neurotransmission – and to reveal if such alterations are reflected at the level of fine structure of the porosome complex.

In the present research we examined the ultrastructure of neuronal porosome complex in cats subjected to continuous white noise that is relevant to the increasing, random noise encountered by humans in today’s environment. The white noise is known to provoke diverse effects on different regions of the brain. Thus, in contrast to hypokinetic stress, constant exposure to such intervention does not provoke significant pathological alterations in the structure of synapses, however, it sabotages the development and normal function of auditory and some other brain regions, and ultimately impair hearing, language acquisition^16,17^, memory performance^18,19^and other cognitive functions. The basis of such alterations should be corresponding abnormalities in neurotransmitter release systems and some other molecular changes, such as Glu and N-methyl-D-aspartate acid receptor 2B, GABA receptors or tau phosphorylation that take place in several regions of the brain, including the auditory system^20-23^. In our study, using EM, we describe porosome complex in two subcortical auditory regions – medial geniculate body (the thalamic structure that serves as the last of a series of processing centers along the auditory pathway from the cochlea to the temporal lobe of the cerebral cortex) and inferior colliculus, the midbrain structure, that acts as the pathway for almost all auditory signals in the body, performs the fundamental role of signal integration, frequency recognition, and pitch discrimination, processes sensory signals to and from the superior colliculus.

## MATERIALS AND METHODS

### Animals 

Adult male cats (14-16 months old), from ordinary vivarial conditions (temperature 20–22°C, humidity 55–60%, light on 07.30–19.30) were used in this study (n = 7 in control and experimental groups). The procedures for handling and caring for the animals were approved by the Animal Care Committee of I. Beritashvili Center of Experimental Biomedicine and are in accordance with current international laws and policies.

### Experimental design

Adults male cat were noise-exposed (1 h, 5-20 kHz, 95-100 dB) and euthanized under anesthesia to obtain brain tissue (n=4). Unexposed age-matching cats were used as control (n=3).

### Electron microscopic examination

Following pentobarbital injection (100 mg/kg), animals to have EM examination of their brains underwent transcardiac perfusion with heparinized 0.9% NaCl, followed by 500 mL of 4% paraphormaldehyde and 2.5% glutaraldehyde in 0. 1 M phosphate buffer (PB), pH 7.4, at a perfusion pressure 120 mm Hg. The brains were removed from skull and placed in the same fixative overnight. The right hemispheric tissue blocks containing *the inferior colliculi (IC)* and the *medial geniculate body (MGB)*were cut into 400 micron-thick coronal slices. Slices were washed in cold 0.1 M PB and kept in 2.5% glutaraldehyde in 0.1 M PB until processing; when processing, the slices were washed in cold PB, post-fixed in 1% osmium tetroxide in cold PB for 2 h and again washed in 0.1 M PB. The IC and GMB were identified with an optical microscope Leica MM AF, cut out from the coronal slices, dehydrated in graded series of ethanol and acetone, and embedded in araldite. Blocks were trimmed and 70–75 nm thick sections were cut with an ultra-microtome Reichert, picked up on 200-mesh copper grids, double-stained with uranyl-acetate and lead-citrate, and examined with a JEM 100 C (JEOL, Japan), HF 3300 (Hitachi, Japan) and Tesla BS 500, Czechoslovakia) transmission electron microscopes. For each case, 120 sections were observed.

### Morphometric analysis of porosome diameter and depth

**Figure 1:**
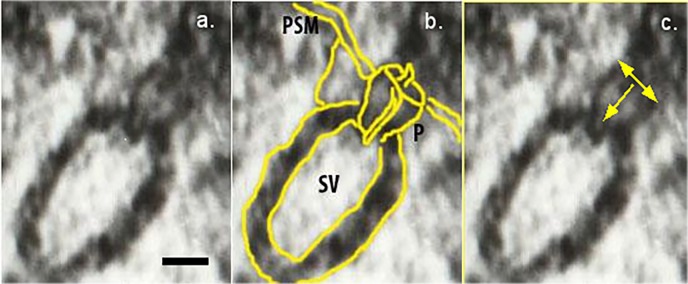
Electron micrograph of a synaptic vesicle docked at the base of a cup-shaped neuronal porosome complex (a) A 50 nm synaptic vesicle (SV) docked at the base of a 15 nm porosome complex (P) at the presynaptic membrane (PSM). Note the central plug of the porosome is clearly visible. (b) For clarity, the outline of the synaptic vesicle and the porosome complex with the central plug is traced using yellow lines. (c) The double-headed arrow represents the diameter of the porosome opening; the single-headed arrow pointing inward represents the depth. Scale Bar = 10 nm.

In order to identify any differences with regard to diameter and depth of porosome complexes in the brain tissue of the cat affected with white noise, a morphometric analysis was performed. A total of 420 synaptic terminals werestudied in the IC (182 synapses) and the MGB (238 synapses) of the cat brain and 178 neuronal porosomes were identified (79 in IC and 97 in MGB). The depth and diameter of these porosomes (**Figure 1**) were measured with “Image J” software and the data are represented in **Table 1**.

**Table 1:** Diameter and depth (in micrometers) of IC and MGB porosomal complex in control and experimental cat brain

N	Inferior Colliculi (IC)				Medial Geniculate Body (MGB)			
	Control		Experiment		Control		Experiment	
	Diameter	Depth	Diameter	Depth	Diameter	Depth	Diameter	Depth
1	0.019	0.012	0.019	0.015	0.016	0.011	0.014	0.013
2	0.017	0.009	0.008	0.014	0.015	0.011	0.018	0.012
3	0.016	0.011	0.012	0.013	0.012	0.013	0.031	0.018
4	0.011	0.011	0.017	0.015	0.012	0.017	0.014	0.008
5	0.011	0.007	0.018	0.016	0.019	0.01	0.011	0.009
6	0.014	0.011	0.018	0.017	0.009	0.011	0.017	0.012
7	0.012	0.015	0.013	0.006	0.016	0.021	0.016	0.013
8	0.015	0.007	0.014	0.01	0.018	0.01	0.015	0.018
9	0.007	0.014	0.016	0.016	0.009	0.006	0.017	0.014
10	0.015	0.016	0.02	0.025	0.014	0.012	0.018	0.01
11	0.017	0.008	0.017	0.008	0.012	0.017	0.015	0.011
12	0.012	0.009	0.014	0.01	0.015	0.011	0.019	0.018
13	0.007	0.007	0.017	0.017	0.013	0.009	0.019	0.014
14	0.011	0.006	0.013	0.008	0.018	0.011	0.011	0.017
15	0.013	0.006	0.018	0.014	0.013	0.008	0.018	0.01
16	0.01	0.008	0.019	0.012	0.017	0.016	0.018	0.012
17	0.018	0.013	0.015	0.01	0.017	0.011	0.011	0.016
18	0.015	0.007	0.008	0.008	0.012	0.016	0.014	0.015
19	0.007	0.007	0.008	0.008	0.014	0.013	0.011	0.007
20	0.014	0.006	0.01	0.007	0.015	0.008	0.014	0.011
21	0.01	0.008	0.015	0.008	0.012	0.012	0.016	0.018
22	0.018	0.015	0.013	0.009	0.011	0.012	0.018	0.015
23	0.018	0.01	0.012	0.009	0.011	0.007	0.014	0.014
24	0.009	0.006	0.017	0.011	0.016	0.012	0.013	0.015
25	0.019	0.011	0.014	0.012	0.018	0.013	0.015	0.015
26	0.013	0.007	0.019	0.009	0.015	0.01	0.015	0.019
27	0.016	0.016	0.017	0.013	0.018	0.01	0.018	0.02
28	0.013	0.016	0.015	0.014	0.01	0.008	0.018	0.014
29	0.018	0.015	0.013	0.011	0.016	0.007	0.018	0.017
30	0.009	0.011	0.014	0.015	0.016	0.012	0.015	0.01
31	0.018	0.011	0.018	0.016	0.011	0.007	0.016	0.008
32	0.018	0.013	0.014	0.01	0.018	0.012	0.016	0.012
33	0.013	0.008	0.009	0.011	0.016	0.009	0.016	0.017
34	0.012	0.013	0.014	0.009	0.019	0.013	0.018	0.019
35	0.018	0.012	0.012	0.015	0.01	0.01	0.01	0.017
36	0.016	0.006	0.019	0.011	0.015	0.009	0.015	0.012
37	0.014	0.011	0.011	0.009	0.018	0.008	0.015	0.006
38	0.021	0.009	0.014	0.012	0.015	0.014	0.011	0.009
39	0.021	0.01	0.012	0.007	0.011	0.007	0.014	0.013
40			0.015	0.012	0.015	0.008	0.019	0.013
41					0.018	0.013	0.018	0.012
42					0.012	0.009	0.019	0.011
43					0.012	0.009	0.017	0.014
44					0.018	0.007		
45					0.019	0.013		
46					0.016	0.007		
47					0.02	0.015		
48					0.014	0.012		
49					0.018	0.012		
50					0.015	0.012		
51					0.018	0.013		
52					0.026	0.015		
53					0.03	0.015		
54					0.02	0.018		

### Statistical analysis

To determine whether the white noise provokes effect on the sizes of neuronal porosome complex diameter and depth, the one-way ANOVA was performed separately in IC and GMB. In the case of significant effect planned comparisons were carried out using *t*-test. The results were presented as a mean ± standard error (SE).

Two way ANOVA test was used to analyze the combined effects of white noise exposure and porosome location in different brain structures. A *p*-value less than 0.05 were considered as statistically significant.

## RESULTS

**Figure 2:**
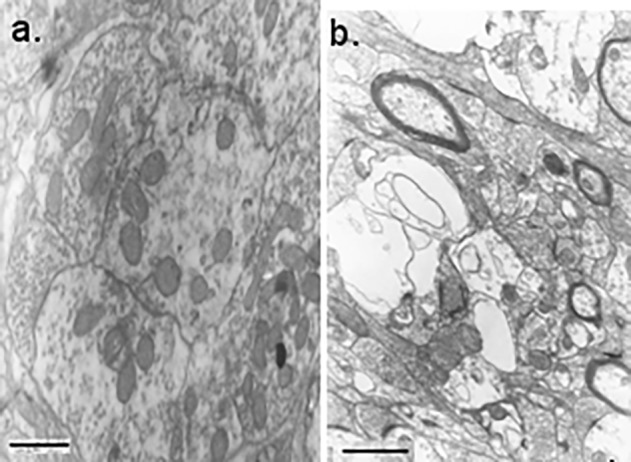
Ultrastructure of MGB in the brain of control (a) and experimental animals (b) (a)Scale bar = 0.5 µm; (b) Scale bar = 0.25 µm.

**Figure 3:**
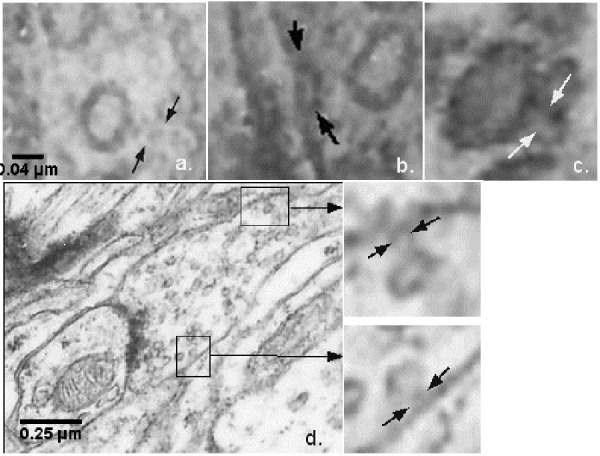
Electron micrograph of neuronal porosome complexes in the cat brain (a, c) Magnified synaptic active zone of an axo-dendritic synapse with 17–19 nm cup-shaped porosome at the presynaptic membrane (black and white arrows) with docked synaptic vesicles (SV) in control IC and MGB; (b, d) vesicle-docked porosome structures (black arrows) in experimental IC and MGB.

According EM studies, continuous white noise provokes several alterations in the synapses of auditory subcortical structures. These alterations are more prominent in GMB (**Figure 2** [b]). Thus, in large presynaptic terminals with polymorphic vesicles (the terminals of specific afferents) and small presynaptic terminals with spherical or flattened vesicles, the number of synaptic vesicles is decreased and the postsynaptic osmiophilia is increased. In some cases the length of synaptic active zone seems to be increased. In several synaptic terminals of experimental animals pathological alterations, such as clustering of synaptic vesicles, swelling, partial vacuolization or degeneration of mitochondria, were observed. In postsynaptic regions numerous vacuoles and some mitochondria are partly vacuolated (**Figure 3** [b,d]). Based on such data, we suggest that as a result of auditory stimulation, in MGB the depletion of some synapses (probably formed by specific auditory afferents), due to their hyperactivation by continuous white noise takes place. Some other synapses (formed by small presynaptic terminals with spherical vesicles) remained unchanged.

According EM studies, continuous white noise provokes several alterations in the synapses of auditory subcortical structures. These alterations are more prominent in GMB (**Figure 2** [b]). Thus, in large presynaptic terminals with polymorphic vesicles (the terminals of specific afferents) and small presynaptic terminals with spherical or flattened vesicles, the number of synaptic vesicles is decreased and the postsynaptic osmiophilia is increased. In some cases the length of synaptic active zone seems to be increased. In several synaptic terminals of experimental animals pathological alterations, such as clustering of synaptic vesicles, swelling, partial vacuolization or degeneration of mitochondria, were observed. In postsynaptic regions numerous vacuoles and some mitochondria are partly vacuolated (**Figure 3** [b,d]). Based on such data, we suggest that as a result of auditory stimulation, in MGB the depletion of some synapses (probably formed by specific auditory afferents), due to their hyperactivation by continuous white noise takes place. Some other synapses (formed by small presynaptic terminals with spherical vesicles) remained unchanged.

As in our previous studies (Okuneva et al., 2012^12,13^, Japaridze et al. 2012^5^) due to the size of neuronal porosome structure (10-18 nm) and the presence of high concentrations of proteins at the presynaptic membrane, it has been difficult to observe these structures in electron micrographs. Consequently, totally 420 synaptic terminals were studied and only 178 porosomes were revealed. Despite these difficulties, their identified profiles have a cup-shaped structure (**Figure 3** [a-d]) and are displayed with or without docked synaptic vesicles in control and experimental brain.

**Figure 4:**
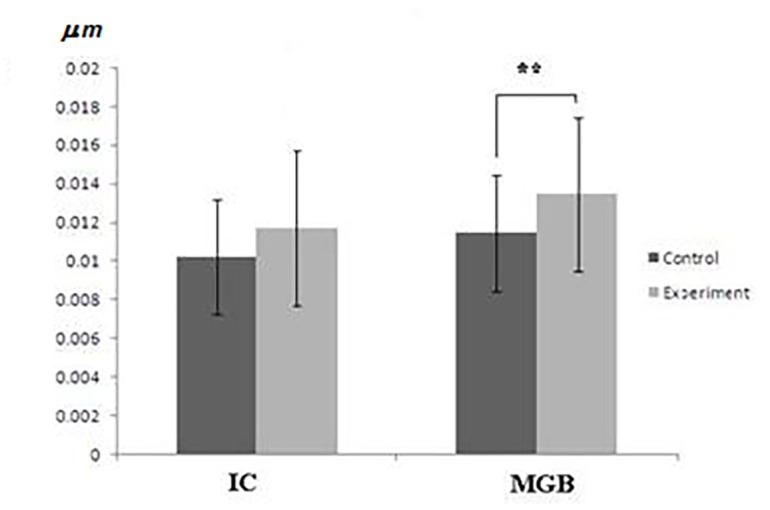
The neuronal porosome depth (mm) in cat brain IC – inferior colliculi, MGB – medial geniculate body; * - *p *< 0.05, ** - *p *< 0.01

**Figure 5:**
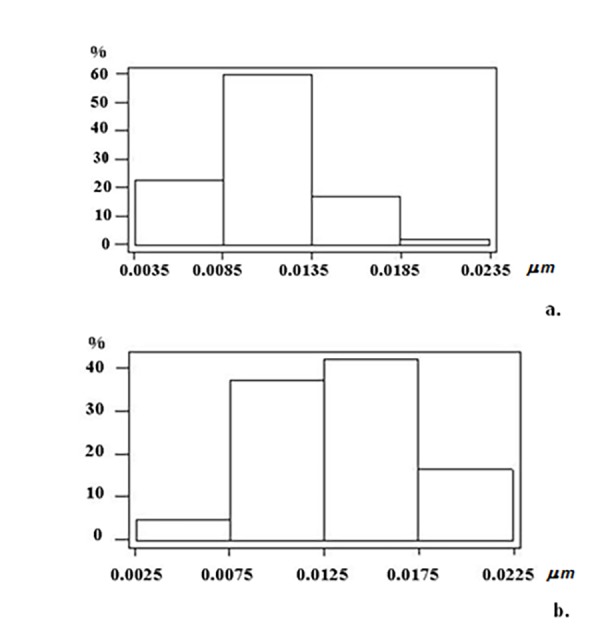
The histograms of neuronal porosome distribution based on the depth value in the synaptic boutons of medial geniculate body (MGB) **a** – control and **b **– experimental group of animals. All measured porosomes are separated by depth’s size and binned by area in 0.0050 µm natural log increment.

According to one-way ANOVA white noise did not affect the opening diameter of porosomes in IC and MGB (*F*=1.955, *p*=0.12), but the depth of porosomal complex is changed significantly (*F*=6.434, *p*=0.0004). Planning comparisons of depth between control and experimental groups of data using two sample *t*-tests revealed that the difference exist only in MGB porosomes (**Table 2**): the mean value of porosome depth in experimental group of data is significantly higher than in MGB control (control 0.01142 ± 0.003 vs. experiment 0.013442 ±0.004*, p *< 0.01) (**Figure 4**). Histograms of porosome distribution in MGB demonstrate, that in control group the main part of porosomes (≈60%) have the depth size 0.0085-0.0135 µm; in experimental group - ≈40% 0.0075-0.0125 µm and >40% 0.0125 – 0.0175 µm. Also ≈15% of neuronal porosomes has depth size ranging 0.00175-0.0225 µm. According to histograms, the difference between control and experimental data arise due to percentage increase of porosomes with depth size ranging from 0.0125 µm – 0.0175 µm and 0.0175 µm – 0.0225 µm in GMB. In all cases a *p *- value threshold is ≤0.05 (**Figure 5**).

**Table 2:** The results of one-way ANOVA and of the two-sample t-test of neuronal porosome depth *F*-variance ratio from one-way ANOVA; *P*-probability.

One-way ANOVA			Unpaired t-test of neuronal porosome depth (µm)			
**Parameters**	**F_(3,177)_**	**P**	**Structure**	**Control**	**Experiment**	***P-value***
**Diameter**	1.955	0.12	IC	0.01021±0.003	0. 01170±0.004	0.062
**Depth**	6.434	0.0004	MGB	0.01142±0.003	0.013442 ±0.004	0.004

To determine whether the two factors – white noise and location of porosome – affect their parameters – diameter of opening and depth – the two-way ANOVA was performed and no significant results were obtained (white noise *F_7,177 _*= 1.03, *p*=0.312; location F_*7, 177 *_= 1.04, *p*=0.310).

Consequently, our results demonstrate that the parameters of porosomes – diameter of opening and depth – are very heterogeneous “dimensions” and the range of their fluctuations (diameter: 12–16 nm, depth: 5–20 nm) are the same in IC of normal cat brain and the brain affected by white noise. The depth of porosomes increases in presynaptic terminals of MGB, but to consider this alteration as the selective effect of white noise exposure on porosomes, allocated in different brain regions, more measurements in more different brain structures of auditory system would be required.

In summary: our results obtained with the morphometric analysis of neuronal porosome complex in brain subcortical auditory areas – MGB and IC – of control cats and cats subjected to continuous white noise revealed the following:

- white noise doesn’t affect the structure of porosome in IC;

- white noise affects porosome structure in MGB, specifically, significant alterations were observed in the depth of porosome;

- as a result of white noise exposure, in MGB the depth of porosome is significantly increased.

## DISCUSSION

In the present EM study we elucidate the effect of continuous white noise on porosome complex structure in two subcortical auditory areas: IC and MGB. The main finding of the study is: continuous white noise does not affect the overall morphology of porosome complex in IC, but provokes alterations in the morphology of the porosome complex in MGB. Specifically, significant increase of porosome depth in this area (MGB) is detected.

While the noise-induced effects within the inner ear have been well investigated, there are only few reports on central noise effects. These effects are mainly contradictory depending on the level of noise, the groups of individuals, their age and brain region^18,19,23^. Thus, the recent computational model, based on the concepts of stochastic resonance and dopamine related internal noise, postulates that a moderate amount of auditive noise benefits individuals in hypodopaminergic states^17,18^. Specifically, noise exposure improves memory performance in children with Attention Deficit Hyperactivity Disorders or inattentive school children, but inhibits performance in high achievers. The conclusion from these studies is that the external auditory noise can restore low dopamine levels and thus, improve cognitive performance. Moreover, it is proposed that the dopamine levels modulate the stochastic resonance effect; this means that low dopamine persons require more noise to obtain stochastic effect^18,20^. Brain regions which are especially sensitive to such effect of noise are mostly dopamine-containing midbrain structures^20^. In contrast, other data demonstrate that white noise provokes prominent negative effect on auditory cortical and subcortical areas and brain regions involved in learning, memory and other cognitive functions^21-27^. Thus, the delay of the functional organization of auditory areas, alterations in different types of memory, cognitive deficits, apoptosis, the metabolic changes, changes in neurotransmitter signaling, receptor function, tau phosphorylation and some other biochemical and molecular modifications were detected^21-24^. Some of such modifications are particularly prominent in the MGB^25,27^.

MGB – prominent cell groups in the postero-inferior aspects of the thalamus, serves as the last of a series of processing stations along the auditory conduction pathway to the cerebral cortex and as the thalamic relay between the IC and the auditory cortex. As to IC, this midbrain structure, the principal auditory center for the body, processes auditory signals from both ears and acts as the pathway for almost all auditory signals in the human body, specifically, performs the fundamental role in signal integration, frequency recognition, or pitch discrimination. Both subcortical structures show comparatively higher rates of metabolic activity than many other areas of the brain. In both regions continuous white noise provokes significant increase of metabolic activity and apoptosis-related pathophysiological changes in time-dependent manner – alterations that are especially interesting for understanding the complex psychoacoustic phenomena of noise-induced hearing loss^26,27^. According to some data, the apoptosis-related and metabolic changes are especially prominent in MGB. The present research also revealed more expressed alterations in the porosome complex in MGB. Functional outcome of such alterations is not well determined. We can discuss such data only on the basis of existing suggestions. Thus, it is predisposed that the position of porosome plug is closely related to the functional state of porosome complex – its opening or closing – which might reflect potential alterations in the neurotransmission^9,10,14^. Therefore, it is very likely that the significant increase of the porosome depth in MGB could be related to the increase of neurotransmission in this subcortical region. Such alterations could be related to the development of compensatory processes in this region, due to its high neuroplasticity (it is well-known that MGB represents the processing station for auditory and some other conducting pathways to cerebral cortex)^25,27,28^.

In our early work we described the morphology of porosome complex in the central nucleus of amygdala of rats subjected to chronic hypokinetic stress. This nucleus is known to be actively involved in the organization of stress-response. Therefore, as a result of chronic stress, prominent pathologies in synapses were described^15^. All these pathologies clearly indicate to the altered neurontransmission in such synapses. But despite such pathologies the morphology of porosome complex remained unchanged^5,14^. In contrast, in the present study alterations in synapses of MGB were much less pronounced; however significant alterations develop in the morphology of the porosome complex. Results from the study demonstrate for the first time, alteration of the neuronal porosome complex in a pathophysiological setting, reflecting its critical role in health and disease.

In conclusion: our results demonstrate that the parameters of porosomes – diameter of opening and depth – are very heterogeneous “dimensions”. In the IC the range of their fluctuation (diameter – 12-16 nm, depth – 5-20 nm) are the same as observed in the normal cats and cats subjected to continuous white noise. In contrast, in the MGB, significant alterations in the morphology of the porosome complex are detected. These defects may reflect specific biochemical changes that result in such morphological changes at the nanometer level within porosomes of a specific region of the brain. Our ongoing studies at the biochemical level will address this issue.

## Bullet Points

- **Neuronal porosomes have a unique structure and are affected by white noise in the auditory subcortical areas. **
- **The significant increase of porosome depth in subcortical auditory area may reflect the alteration in neurotransmission.**
